# The Impact of Adverse Childhood Experiences on Youth’s Mental Health: What is the Role of Family Resilience?

**DOI:** 10.1007/s40653-026-00966-4

**Published:** 2026-08-27

**Authors:** Jusuk Song, Hyuncheol Yoon, Mihee Woo, Isak Kim

**Affiliations:** 1 Wheaton College - Illinois, Wheaton, USA; 2 University of Florida, Gainesville, USA; 3 Thomas Jefferson University, Philadelphia, USA; 4 University of Iowa, Iowa City, USA

**Keywords:** Adverse childhood experiences, Mental health, Internalizing problem, Externalizing problem, Family resilience

## Abstract

Given that many adverse childhood experiences (ACEs) are family-contextual and that family processes shape youth mental health, the current study examined the role of family resilience in the relationship between ACEs and major mental health outcomes among youth aged 6–17 years. We employed the data from 2022 National Survey of Children’s Health in the United States. Structural equation modeling was estimated using the mean- and variance-adjusted weighted least squares estimator (WLSMV) given the categorical nature of the data. The study results revealed a good model fit (CFI = 0.974, TLI = 0.969, RMSEA = 0.028, and SRMR = 0.044). Statistically significant pathways were found between ACEs and mental health outcomes (*β* = 0.50, *p* < .001), ACEs and family resilience (*β =* − 0.19, *p* < .001), and family resilience and mental health outcomes (*β =* − 0.11, *p* < .001). Additionally, family resilience showed a significant indirect effect on the relationship between ACEs and mental health outcomes (*β =* 0.02, *p* < .001). These findings highlight the potential role of family-oriented approaches for effective trauma prevention and youth mental health interventions.

**Supplementary Information:**

The online version contains supplementary material available at https://doi.org/10.1007/s40653-026-00966-4.

## Introduction

The mental health crisis among youth is increasingly recognized, with recent statistics highlighting an urgent need for addressing major mental health problems. Data from 2021 to 2022 National Survey of Children’s Health indicate that attention-deficit/hyperactivity disorder (ADHD), behavioral problems, anxiety, and depression are among the most commonly diagnosed mental, emotional, and behavioral conditions among children aged 3–17 years. Prevalence estimates show that approximately 7.0 million children (11.4%) have been diagnosed with ADHD, 4.3 million (7.0%) with behavioral problems, 6.1 million (10.0%) with anxiety, and 2.4 million (4.0%) with depression (Centers for Disease Control and Prevention, 2024).

Prior research has documented that exposure to adverse childhood experiences (ACEs), including household dysfunction and contextual adversity during childhood, is associated with an increased risk of these mental health problems (Andersen, 2021; Finkelhor et al., 2015; Graf et al., 2021). It is evident that ACEs in early life erode a family’s capacity to bounce back from adverse environments, which can play a critical role in understanding the mechanisms through which ACEs are linked to mental health consequences in youth (Scully et al., 2020). Indeed, many ACEs, such as parental divorce, exposure to domestic violence, economic hardships, and growing up in a household with severe mental illness, are inherently embedded in family dynamics (Centers for Disease Control and Prevention, 2024; Narayan et al., 2021). Accordingly, family resilience plays a critical role in understanding and responding to these adverse events (Okwori, 2022). By examining the associations among ACEs, family resilience, and mental health outcomes, researchers and professionals can gain a clearer understanding of how these factors co-occur and identify relevant patterns that may inform the development of intervention strategies.

### Youth Mental Health

Youth mental health is commonly conceptualized as internalizing and externalizing problems, which serve as key indicators of psychopathology in the youth population (Achenbach & Rescorla, 2001; Heleniak et al., 2016). This characteristic reflects the widely supported two-domain structure of child psychopathology (Achenbach & Rescorla, 2001; Cosgrove et al., 2011).

At a broader level, research has demonstrated that common mental health disorders can be classified into higher-order category of internalizing and externalizing problems (Caspi et al., 2014; Krueger, 1999). Empirical evidence further supports this structure, with studies showing that a two-factor model provides a better fit to adolescents’ mental health data than a single-factor model (Cosgrove et al., 2011). These domains often co-occur, and prior research suggests substantial overlap across different mental health conditions (Achenbach et al., 2016; Caspi et al., 2014). However, studies examining the relationship between ACEs and youth mental health have frequently focused on specific disorders or domains in isolation, limiting a more comprehensive understanding of overall mental health risk (Elmore & Crouch, 2020; Hertz, 2020; Jackson et al., 2023; Kerker et al., 2015).

To address this gap, the present study examines youth mental health in a more integrated manner by considering both internalizing and externalizing problems simultaneously. Consistent with established models of child psychopathology, anxiety and depression are typically conceptualized as internalizing problems (Khanijahani & Sualp, 2022; Lew & Xian, 2019; Liu et al., 2011, whereas ADHD and conduct-related problems are commonly conceptualized as externalizing problems (Atherton et al., 2020; Danielson et al., 2018; Shaffer et al., 2000). Although psychopathology is generally understood to lie along a continuum, clinically meaningful decisions in research and practice often rely on threshold-based classifications to identify youth with clinically significant mental health problems (Kotelnikova et al., 2026). Framing youth mental health in this way allows for a more comprehensive examination of clinically meaningful mental health difficulties and their associations with contextual risk factors such as ACEs.

### The Relationship Between ACEs and Youth’s Mental Health

The term ACEs, coined by Vincent Felitti and colleagues in their seminal paper in 1998, refers to potentially traumatic events that children experience before age of 18 (Felitti et al., 1998). The NSCH-ACE questionnaire builds upon the original ACEs framework (Felitti et al., 1998) by retaining core indicators such as parental divorce, death of a parent, mental illness within the household, parental substance use, domestic violence, and parental incarceration. In addition, it broadens the scope of adversity to include experiences such as economic hardship, witnessing community violence, and perceived discrimination (Finkelhor et al., 2015). These items reflect family-embedded adversities that are most proximal to family functioning (Bethell et al., 2017; Kim et al., 2024). Additionally, given the co-occurring and cumulative nature of multiple ACEs (Hughes et al., 2017), a comprehensive understanding of ACEs is more appropriate than treating each ACE as a discrete event. According to the Centers for Disease Control and Prevention (2024), the prevalence of ACEs in the United States indicates that 64% of adults have experienced at least one ACE before age 18, and 17.3% report having experienced four or more types of ACEs. Among youth aged 6–17 years, nearly 61.6% have experienced one ACE, with 23% reporting exposure to two or more ACEs (Hertz, 2020).

The associations between ACEs and youth’s mental health, including externalizing and internalizing problems, have also been extensively documented (e.g., Bai & Repetti, 2015; Hertz, 2020; Hunt et al., 2017; Jackson et al., 2023). Hunt et al. (2017) reported that exposure to multiple ACEs from birth through age 5, particularly among Black children whose mothers had lower education levels, was associated with both externalizing and internalizing problems as well as increased likelihood of an ADHD diagnosis in middle childhood. Notably, children who experienced household-related ACEs, such as socioeconomic hardship, parental divorce, or familial mental illness, had significantly higher odds of ADHD (Brown et al., 2017). Kerker et al. (2015) underscore a strong association between the number of ACEs and negative outcomes in early childhood mental health, such as anxiety, depression, attention problems, and particularly for children aged 18 to 71 months involved in the child welfare system. Additionally, a growing body of empirical evidence supports the association between ACEs and children’s internalizing problems (Elmore & Crouch, 2020; Khanijahani & Sualp, 2022; Lee et al., 2020; Lew & Xian, 2019). Taken together, a substantial body of research demonstrates that higher exposure to ACEs is associated with an increased likelihood of mental health problems among youth. However, it remains important to examine familial factors that may help contextualize this relationship given that ACEs are strongly shaped by proximal influences, particularly within the family environment (Kalmakis & Chandler, 2014).

### Family Resilience as a Potential Mediator

Family-level processes during or after ACEs are likely to play a pivotal role in shaping how these experiences influence youth mental health. Accordingly, our study focuses on family resilience, defined as a family’s ability to withstand, adapt to, and recover from crises by enhancing both individual and collective resilience (Walsh, 1996). Specifically, under Walsh’s (1996) framework, belief systems (e.g., positive outlook, collective hopefulness) and communication and problem-solving processes (e.g., collaborative decision-making, coping strategies) are highlighted as centrally operative in active crisis responses. Indeed, family resilience, often described as family cohesion, warmth, and shared problem solving in times of adversity, has been identified as a key protective mechanism that explains the effects of child abuse and other adverse events in childhood (Luthar & Zigler, 1991; Masten et al., 1990).

Within the developmental systems framework, adverse experiences in early life can influence the entire family system over time, including individual members, relationships, and the family as a unit. In turn, these experiences impact the formation of family resilience and may accelerate the adaptive or maladaptive family processes (Walsh, 2016). Family-level ACEs themselves can reflect risk factors characterized by the absence of essential components needed to build family resilience, such as financial insecurity, chronic illness or disability among family members, and inadequate conflict management within a family unit (Walsh, 1996, 2016). Moreover, family system theory suggests that a single family member can disrupt overall family functioning, as families are interdependent emotional systems (Bowen, 1978). Within these frameworks, ACEs such as domestic violence or living with a person who has mental health problems or substance use issues may hinder the development of resilience in a family unit, serving as indicators of broader family dysfunction. For instance, parental divorce can lead to multiple challenges among family members and domains, including pre-divorce conflict, separation, custody arrangements, and changes in parent-child relationships (Greene et al., 2012). Prior research has consistently exhibited that exposure to ACEs is associated with impairment of family-level adaptive processes, including lower levels of family resilience at both individual and familial levels (e.g., Bethell et al., 2019; Scully et al., 2020). These findings suggest that early life adversity may interrupt families’ capacity to effectively cope with stressors and mobilize resources to respond to adversity.

Consistent with this literature, prior studies have documented robust associations between family-level processes and youth’s internalizing and externalizing problems, including depression, ADHD symptoms, and behavioral problems (Balistreri & Alvira-Hammond, 2016; Choksomngam et al., 2022; Du & Kim, 2020; Huang et al., 2022; Lansford et al., 2006). Although family functioning has been widely investigated as a protective factor against ACEs in prior research, this construct primarily captures routine interaction patterns and general family climate, rather than families’ adaptive responses under conditions of stress (Sangalang et al., 2017; Scully et al., 2020). Family resilience more directly reflects how families respond to and recover from adversity, making it particularly relevant for understanding ACE-related processes.

A growing body of empirical evidence further supports the relevance of family resilience for youth’s mental health outcomes. Specifically, higher levels of family resilience have been associated with lower internalizing and externalizing problems, including depression, ADHD symptoms, and behavioral difficulties (Cheng et al., 2024; Song et al., 2021; Uddin et al., 2020). Additionally, a recent meta-analysis demonstrated a medium-sized positive association between family resilience and positive mental health outcomes (Cheng et al., 2024). Taken together, the existing literature suggests that family resilience may serve an important explanatory role in linking ACE exposure to youth internalizing and externalizing mental health outcomes.

### Present Study

The aims of this study are twofold. First, most prior studies have not adequately addressed internalizing and externalizing conditions comprehensively as outcome variables of ACEs. By employing higher-order confirmatory factor analysis (CFA) to evaluate the statistical adequacy of theoretically informed structures, this study seeks to provide a comprehensive model of a range of mental health outcomes and examine their associations with ACEs. Second, previous research has predominantly viewed family factors as buffers, considering them as moderating variables that offer protection against the adverse effects of ACEs on youth’s mental health. However, this perspective may overlook the formative role of household-level ACEs in shaping family processes. Consistent with family systems theory and Walsh’s developmental systems framework, adverse experiences occurring within the household context, such as parental divorce, death, domestic violence, or economic hardships, can disrupt the development and maintenance of family resilience. Building on this theoretical foundation, the present study addresses a critical gap by focusing on family resilience as a potential mediator in the association between ACEs and mental health outcomes, conceptualizing ACEs as undermining family resilience, which, in turn, is associated with poorer mental health among youth. We posit that weakened family resilience as a result of ACEs may explain why some children exposed to high levels of adversity are at increased risk for developing mental health problems.

## Method

### Data and Sample

The public dataset used in this study is 2022 National Survey of Children’s Health (NSCH). The NSCH survey, conducted by the U.S. Census Bureau, consists of a cross-sectional self-reported or caregiver-reported questionnaires for the noninstitutionalized 0 to 17 years old children in the United States. The raw dataset included survey responses for children aged 0–17 years. Out of 54,103 cases, a total of 34,362 children aged 6–17 years were selected as the final analytic sample. Missing data were addressed using multiple imputation in R via the MICE package, under the assumption that data were missing at random (MAR), as it provides less biased parameter estimates compared to listwise deletion and helps preserve statistical power.

The study participants consisted of school-aged children in the United States (*M* = 11.94, *SD* = 3.53). Children aged 6–11 years accounted for 44.6% of the sample (*n* = 15,334), whereas those aged 12–17 years comprised 55.4% (*n* = 19,028). With regard to gender, 51.9% of participants were male (*n* = 17,830) and 48.1% were female (*n* = 16,532). Regarding participants’ race/ethnicity, the majority of participants identified as White (*n* = 26,193, 76.2%), while 23.8% identified as Black, Indigenous, or People of Color (BIPOC; *n* = 8,169). The BIPOC group included Black or African American (*n* = 2,570, 7.5%), American Indian or Alaska Native (*n* = 374, 1.1%), Asian (*n* = 2,223, 6.5%), Native Hawaiian and Other Pacific Islander (*n* = 227, 0.7%), and Two or More Races (*n* = 2,775, 8.1%). In terms of parental highest education level, less than high school (*n* = 1,372, 4.0%) and high school diploma or vocational diploma (*n* = 5,826, 17.0%) together accounted for 21.0% of the sample, whereas 79.1% hadmore than high school (*n* = 27,164, 79.1%). The participants reported family structure and family poverty level (FPL) as follows: single parent or grandparent or other family households (*n* = 9,316, 27.1%), and two-parent households (*n* = 25,046, 72.9%); 0–99%FPL (*n* = 4,474, 13.0%), 100–199%FPL (*n* = 5,616, 16.3%), 200–399%FPL (*n* = 10,032, 29.2%), and 400%FPL or above (*n* = 14,240, 41.4%).

### Measures

#### Mental Health Outcomes

Mental health outcomes were modeled as a higher-order latent construct indicated by two first-order latent variables (i.e., externalizing problems and internalizing problems). These first-order latent variables were, in turn, indicated by a project-developed four binary observed items including, ADHD, behavioral problem, depression and anxiety. Specifically, ADHD and behavioral problem items were specified as indicators of the externalizing problems latent variable, whereas depression and anxiety items were specified as indicators of the internalizing problems latent variable. Together, the externalizing and internalizing problem constructs constituted the higher-order latent variable representing overall mental health outcomes.

A higher-order CFA is employed to capture the underlying structure of externalizing and internalizing problems. Unlike multilevel CFA, which requires hierarchically nested data, higher-order CFA can be applied for theory testing when two or more related factor structures are theoretically supported (Brown, 2015). The distinction between externalizing and internalizing problem domains is well established in the literatures as a framework for organizing a broad range of mental health symptoms (Achenbach et al., 2016).

Each observed item was assessed using this question, “Has a doctor or other health care provider EVER told you that this child has [condition]?” Responses were dichotomous (1 = Yes, 0 = No). Although not the primary criterion for categorical indicators, it was reported to provide supplementary evidence of reliability. Internal consistency reliability was acceptable, with Cronbach’s α = 0.68 for overall mental health outcomes, α = 0.68 for internalizing problems, and α = 0.65 for externalizing problems.

#### Adverse Childhood Experiences

ACEs are modeled as a latent variable indicated by nine observed binary ACEs items. The items of ACEs used in this study were drawn from the 2016 National Survey of Children’s Health (NSCH) and represent a modified version of the ACEs module originally included in the 2011 NSCH.

The NSCH-ACEs items were originally derived from Behavioral Risk Factor Surveillance System (BRFSS) developed by a technical expert panel. These items reflect a revised conceptualization of ACEs based on the original framework proposed by Felitti et al. (1998), while being tailored for use in a nationally representative pediatric sample.

In this study, ACEs consist of nine items which address Economic hardships (ACE1), Parental Divorce (ACE3), Parental Death (ACE4), Parental Incarceration (ACE5), Domestic violence (ACE6), Neighborhood violence (ACE7), Living with the household who has mental health disorders (ACE8), Living with the household who has drug use problems (ACE9), and Racial discrimination (ACE10).

Although some ACEs items exhibited standardized loadings below conventional thresholds (i.e., 0.50; Hair et al., 2010), they were retained in the full indirect-effect model due to their theoretical importance in representing distinct domains of childhood adversity. Additionally, standardized factor loading scores were consistent with prior ACE measurement literature, ranging from 0.40 (racial discrimination) to 0.87 (domestic violence; Jensen et al., 2024). This variability reflects the heterogenous nature of ACEs indicators and supports the conceptualization of ACEs as a multidimensional construct (Afifi et al., 2020).

Considering the statistical benefits of using latent variables (Bethell et al., 2017; Weller et al., 2022), the current study created latent variables to secure model fits and reduce the measurement error, aligning with the prior studies (Tan et al., 2017; Weller et al., 2022). Our research congregated nine ACEs items into one latent item. (See Supplement 1 for item descriptions and factor loading of study variables). Although not the primary criterion for categorical indicators, it was reported to provide supplementary evidence of reliability. The internal consistency of the scale was acceptable (Cronbach’s α = 0.68).

#### Family Resilience

Family resilience was measured using a theoretically grounded composite index from the National Survey of Children’s Health (NSCH), widely used in prior research. The measure consists of four items assessing family communication, problem-solving, perceived strengths, and optimism during adversity (Bethell et al., 2017) and is conceptually consistent with Walsh’s family resilience framework (Walsh, 2016). The items were introduced with the stem question: “When your family faces problems, how often are you likely to do each of the following?” The four items (FAM1- FAM4) included: FAM1, “How often does your family talk together to solve problems?”; FAM2, “How often does your family work together to solve problems?”; FAM3, “How often does your family drawing the strengths of the family?”; and FAM4, “How often does your family staying hopeful?” Participants responded to four items on a four-point Likert scale as follows: (0) “None of the time”, (1) “Some of the time”, (2) “Most of the time”, and (3) “All of the time.” The internal consistency of the scale was acceptable (Cronbach’s α = 0.90).

#### Covariates

Gender, age, parental highest education and family poverty level (FPL) were included as covariates in the final structural equation modeling (SEM) model to account for potential sociodemographic confounding in the associations among ACEs, family resilience, and mental health outcomes.

### Data Analyses

The present study examines the indirect effects of family resilience in the relationship between ACEs and mental health outcomes (internalizing and externalizing problems). While we establish the necessary statistical relationships to test for mediation (Baron & Kenny, 1986), we acknowledge that true mediation requires longitudinal or experimental data to infer causality (Maxwell & Cole, 2007). Given that this study is cross-sectional, our findings represent associational mediation rather than causal mediation. This approach is commonly used in SEM to explore indirect effects when longitudinal data is unavailable (Shrout & Bolger, 2002). To assess indirect-effect models, we used diagonally weighted least squares (DWLS) method for estimating latent response variables.

The current study involved the procedures to transform binary variables into latent variables (i.e., ACEs and mental health outcomes). Specifically, mean-and variance-adjusted weighted least squares (WLSMV) method, known as the robust variant of Weighted Least Squares method in Mplus 8.1 was employed. WLSMV is a robust estimator commonly used for modeling categorical variables as latent constructs when samples size is sufficiently large (Muthen & Kaplan, 1985, 1992). Prior research using NSCH dataset also this method to improve statistical rigor such as addressing measurement errors and goodness-of-fit of the suggested model (Tan et al., 2017). The WLSMV approach was used to estimate indirect effects with bootstrapped confidence intervals to determine whether family resilience significantly accounted for the association between ACEs and mental health outcomes.

In particular, multiple data analyses were conducted. First, preliminary analyses assessed potential multicollinearity, including tolerance values, variance inflation factors (VIFs) using collinearity diagnostics, and bivariate correlation coefficients among independent variables. Additionally, normality was assessed through the skewness and kurtosis standard for observed continuous variables.

Second, two-step approach was utilized to evaluate the measurement model and structural model (Anderson & Gerbing, 1988). In the first step, we conducted higher-order CFA to check the model fit of our suggested indirect effect models and associations between ACEs, family resilience, and mental health outcomes. Sensitivity analysis was conducted to compare the fit indices of full model and reduced model. As a second step, we conducted structural equation modeling to investigate the regression coefficients of each pathway and indirect effect of family resilience between the relationship of ACEs and mental health outcomes. To obtain robust estimates under potential non-normality, 1,000 bootstrap resamples were generated to assess the stability and confidence intervals of the model parameters.

## Results

### Preliminary Analyses and Descriptive Statistics

Preliminary analyses indicated that tolerance values ranged from 0.23 to 1.00, exceeding the recommended threshold of 0.2. VIF values ranged from 1.00 to 4.28, also satisfying the acceptable standard of 5. The absolute values of correlations among all observed study variables ranged from 0.01 to 0.76, except for the correlation between FAM1 and FAM4 (*r* = .84). Taken together, these indicators suggest that multicollinearity was not present among the independent variables and covariates. Additionally, the normality of continuous variable (i.e., family resilience) was assessed and met the recommended standards, with skewness ranging from − 1.03 to -0.83 within the acceptable range of ± 2 and kurtosis ranged from 0.06 to 0.53 within the acceptable range of ± 3 (Kline, 2011). Descriptive statistics for study variables and their subcategories are presented in Table 1.

**Table 1:** Descriptive analysis results of mental health, ACEs, and family resilience

	Yes			No		
	*N* (%)			*N* (%)		
Mental Health						
ADHD	5,267 (15.3)			29,095 (84.7)		
Depression	3,087 (9.0)			31,275 (91.0)		
Anxiety	6,173 (18.0)			28,189 (82.0)		
Behavioral Problem	3,684 (10.7)			30,678 (89.3)		
ACEs						
Economic hardships	4,173 (12.1)			30,189 (87.9)		
Parental Divorce	9,232 (26.9)			25,130 (73.1)		
Parental Death	1,391 (4.0)			32,971 (96.0)		
Parental Incarceration	2,408 (7.0)			31,954 (93.0)		
Domestic violence	2,088 (6.1)			32,274 (93.9)		
Neighborhood violence	1,566 (4.6)			32,796 (95.4)		
Living with the household who has mental health disorders	3,882 (11.3)			30,480 (88.7)		
Living with the household who has drug use problems	3,928 (11.4)			30,434 (88.6)		
Ethnic/Racial Discrimination	1,670 (4.9)			32,692 (95.1)		
	N	M	SD		Skewness	Kurtosis
Family Resilience						
FAM1	34,362	2.40	0.69		-0.85	0.06
FAM2	34,362	2.47	0.67		-1.03	0.53
FAM3	34,362	2.47	0.62		-0.83	0.09
FAM4	34,362	2.42	0.68		-0.87	0.15

### Measurement model

To confirm the construct validity of the three measures used in this study, the measurement model was evaluated using CFA with 1,000 bootstrapping. The goodness-of-fit statistics of the model reported the construct validity in general. In specific, the measurement model showed good model fit, with CFI = 0.974, TLI = 0.969, RMSEA = 0.028 (95% CI = 0.027–0.029), and SRMR = 0.044.

Additionally, factor loadings of all observed items ranged from 0.368 to 0.874 (see Supplement 1 for full details). Sensitivity analyses indicated minimal differences between the full and revised models (ΔCFI = 0.001, ΔTLI = 0.002, ΔRMSEA = 0.004). The revised model, which excluded two items (i.e., ACE4 and ACE10) demonstrated slightly worse fit compared to the full model (CFI = 0.973, TLI = 0.967, RMSEA = 0.032; 95% CI = 0.031–0.033, and SRMR = 0.044). Given this pattern, the full model was retained as the final model.

All correlation coefficients between latent variables are statistically significant (*p* < .001) as follows; ACEs and mental health outcomes (*r* = .57), ACEs and family resilience (*r* = -.21), and family resilience and mental health outcomes (*r* = -.24).

### Structural Model

**Fig. 1:**
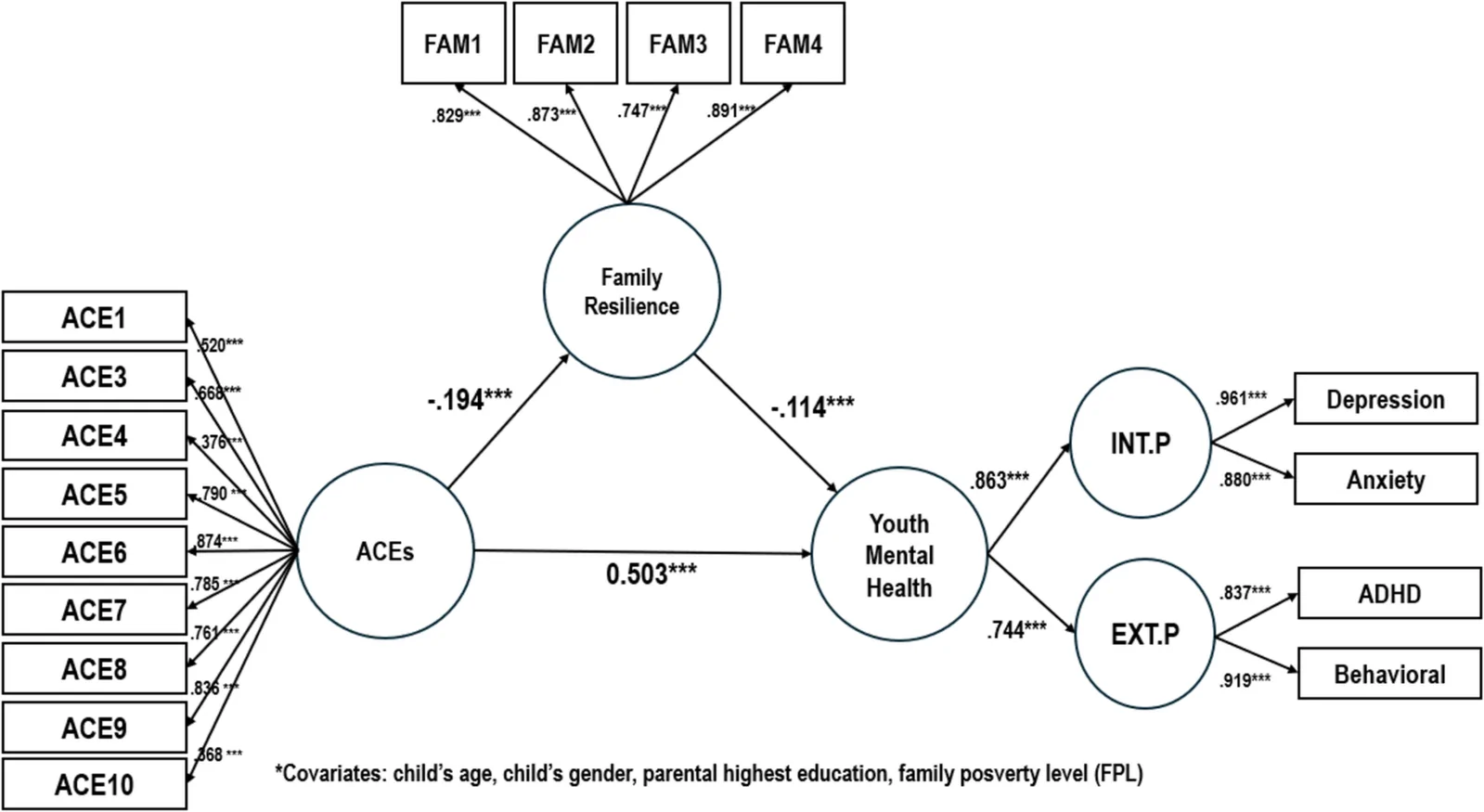
The results of the structural equation model. INT.P = internalizing problems; EXT.P = externalizing problems; Behavioral = behavioral problems

The model showed an acceptable overall fit, with CFI = 0.897, TLI = 0.879, RMSEA = 0.046, and SRMR = 0.093. The fit indices were considered adequate given the overall pattern of model fit, although some indices were below conventional cutoff criteria. Figure 1 presents the results of the structural equation model with 1,000 bootstrap resamples, examining the associations among ACEs, family resilience, and mental health outcomes while controlling for child age and gender, parental education, and family poverty level. The regression coefficients of ACEs to mental health outcomes (*β* = 0.50, *p* < .001), ACEs to family resilience (*β =* -0.19, *p* < .001), family resilience to mental health outcomes (*β =* -0.11, *p* < .001) were reported as statistically significant regression pathways (Table 2). Additionally, the study results indicated that family resilience significantly showed indirect pathway between ACEs and mental health outcomes (*β =* 0.02, *p* < .001). The study results indicate that ACEs have both direct and indirect associations with mental health outcomes through family resilience. Specifically ACEs were associated with lower family resilience, which was subsequently associated with adverse mental health outcomes. These findings supportmodel with both direct and indirect effects.

**Table 2:** Direct, indirect, and total effects

	b	S.E.	Z	95% CI	β
ACEs → Mental Health Outcomes	0.98	0.03	29.38^***^	0.91, 1.05	0.50
ACEs → Family Resilience	-0.24	0.01	-22.78^***^	-0.26, -0.22	-0.19
Family Resilience → Mental Health Outcomes	-0.18	0.01	-13.49^***^	-0.21, -0.15	-0.11
ACEs → Family Resilience → Mental Health Outcomes	0.04	0.003	12.56^***^	0.04, 0.05	0.02

*** *p* < .001. 1,000 bootstrapping

## Discussion

ACEs have a detrimental impact on the mental health of children and adolescents, often leading to both internalizing and externalizing problems. These issues are associated with mental health challenges and lower family resilience, which may in turn be linked to more pronounced effects of ACEs. Given the small magnitude of the indirect effects (*β =* 0.02), the findings suggest potential mechanisms through which mental health problems among youth are understood in relation to ACEs and a family resilience within the familial-level contexts. Importantly, this indirect effect remained significant after controlling for individual- and family-level covariates (e.g., child age and gender, parent education, and family poverty level). This finding underscores that family resilience can still be the important context for understanding youth mental health given that childhood traumatic experiences predominantly occur in the family contexts (Bethell et al., 2017). Therefore, investigating significant associations among ACEs, family resilience, and children’s mental health outcomes, with highlighting the roles of family resilience statistically linking ACEs to mental health indicators, is meaningful to better understand mechanisms of ACEs on mental health outcomes.

Family resilience as a potential mediator reflects possible pathways that ACEs can disrupt both individual and collective coping mechanisms within the family system. Many ACEs at the household level, such as parental mental health challenges or economic hardship, can be negatively associated with family resilience, potentially contributing to the impaired family cohesion and functioning, difficulties with family adjustment, and heightening risks of emotional distress among family members (Family Stress Model; Conger & Donnellan, 2007). This emotional distress, along with increased interpersonal conflicts and poor-quality parenting, may increase the likelihood of damaging effects on their children’s developmental trajectories (Sandler et al., 2013; Sarrazin & Cyr, 2007). Additionally, the diminished family resilience can restrict the family’s ability to foster healthy emotional regulation and communication skills in children, leading to greater risks of developing internalizing and externalizing problems (e.g., Schrodt et al., 2008; Zimmer-Gembeck et al., 2022). These explanations reiterate the need for multi-layered interventions that target adolescents, parents, and the broader family system to effectively mitigate the impact of ACEs on youth mental health outcomes.

The role of family resilience provides a broader perspective to capture both ACEs and children’s mental health problems. Specifically, a dual approach framework, that emphasizes both direct associations of ACEs and indirect associations involving family resilience, helps integrate systemic and socioecological perspectives on children’s mental health. This notion is also aligned with the concept of the intergenerational transmission of mental health and psychiatric risk (Van Santvoort et al., 2015), which highlights how adverse and dysfunctional family environments (e.g., child lived with anyone who was mentally ill, suicidal, or severely depressed) are linked to children’s mental health outcomes. Within such environments, emotional and psychological processes within families have been shown to be associated with children’s mental health and overall well-being (Loeber et al., 2009; Van Santvoort et al., 2015).

Furthermore, our findings can be understood in light of prior research. For example, Wang et al. (2023) demonstrated that family resilience statistically mediated the association between ACEs, particularly emotional and behavioral abuse and children’s emotional and behavioral problems. Consistent with this perspective, economic hardship, as an ACE, may be embedded within adverse family contexts that are associated with poorer mental health outcomes (Weller et al., 2022). In other words, patterns of adversity and poor mental health within family environments can be transmitted across family members and associated with low levels of family resilience. Reduced family resilience, in turn, may limit families’ capacity to respond to ACE-related challenges in a coordinated and effective manner, leading to deterioration in children’s mental health over time.

Importantly, the present study extends this literature by adopting a more methodologically rigorous approach. Specifically, we modeled ACEs, mental health outcomes, and family resilience as latent constructs reflecting their multidimensional structure and capturing their co-occurring nature at the level of observed indicators, while accounting for measurement error across all constructs. In addition, we controlled for relevant individual- and family-level covariates, thereby providing a more conservative test of the proposed associations.

Considering these dynamics, as we discussed earlier, dual approaches for addressing children’s mental health problems can be crucial in providing effective mental health services to children in need because adverse effects are not only directly tied to children’s mental health concerns but are also indirectly linked to families’ complex situations, relationships, and dynamics that influence the children’s externalizing and internalizing problems.

## Clinical Implications

ACEs should not overshadow the potential for family resilience. Instead, researchers highlighted the importance of targeted interventions that foster and build upon this resilience (Webster et al., 2024). Our findings underscore the value of engaging in meaningful conversations with families about their dynamics and sources of strength. Drawing from Traumatic System Theory (Fitzgerald et al., 2020; Whitechurch & Constantine, 1993), which examines trauma through the lens of family systems, and the Resilience Process framework (Bai & Repetti, 2015), effective interventions should address both individual and family-level factors.

At the individual level, interventions should explore how family factors directly impact the child’s mental health and development. Building resilience through parent training programs can improve daily family interactions and reduce the negative effects of ACEs (Bai & Repetti, 2015). In the program, parents can enhance their coping skills, which foster protective factors to meet each child’s specific needs and increase resilience of both parents and a child. Additionally, focusing on trauma-informed approaches that recognize the emotional toll of ACEs is essential. Mental health professionals should help children develop emotional regulation strategies and equip them with tools to manage stress. This can include practices such as mindfulness techniques, cognitive-behavioral interventions, and psychoeducation on how trauma affects behavior and emotions.

At the family level, behavioral monitoring can offer valuable insights for mental health professionals, which facilitate real-time feedback that families can track their thoughts, emotions, and behaviors (Chorpita et al., 2010). This family-level intervention can be particularly useful in addressing strained family processes that have been disrupted by ACEs. Furthermore, parent-child interaction interventions, designed to enhance communication and foster positive interactions, can significantly improve family dynamics (Thomas & Zimmer-Gembeck, 2007). By focusing on communication patterns and conflict resolution strategies, health professionals can help rebuild trust and foster emotional connections within families. Addressing family-focused protective factors (Bethell et al., 2016), such as nurturing relationships and emotional support, should be central to interventions aimed at strengthening family resilience, resultantly reducing the harmful effects of ACEs. Health professionals should also ensure that interventions respect and incorporate a family’s cultural and personal preferences, as resilience-oriented, culturally sensitive approaches have been shown to reduce stress, foster hope, and enhance coping strategies (Walsh, 2021; Rezaei Sharif et al., 2020).

As Masten (2015) emphasizes, family resilience is intertwined with broader systems, underscoring the need for a comprehensive approach to mental health that extends beyond the individual or family unit. Community-based interventions are crucial not only for addressing the systemic challenges that families face, such as parental divorce, economic struggles, and household mental health or substance use issues, but also for preventing ACEs by offering early support and resources. By providing timely access to financial aid, housing stability, and mental health services, communities can reduce the risk factors that contribute to ACEs, thereby lessening their long-term impact. Our data suggests that there is considerable room for improvement in community services designed to prevent and mitigate these challenges. Viewing these issues through a broader societal lens can help health professionals and policymakers identify how enhancing community resources, such as social support networks, mental health programs, and economic assistance, can alleviate pressures on families, strengthen resilience, and provide vital support. An integrated approach considers not only the family’s immediate struggles but also their place within larger societal contexts, such as economic hardship and access to mental health services, which are critical for preventing ACEs and supporting family resilience.

## Limitations and Future Directions

Several limitations of the present study should be acknowledged, each of which provides important directions for future research. First, although one of our study’s strengths involves a comprehensive approach to conceptualizing youth mental health by including both internalizing and externalizing problems, our model does not specifically demonstrate the associations with specific mental health conditions. While broad-band groupings for mental health help explain general mental health issues, narrow-band categories included in our model (e.g., ADHD, anxiety) are also necessary for a more nuanced understanding. For instance, within externalizing problems, it would be valuable to differentiate between aggressive behaviors with or without physical violence (Achenbach et al., 2016). Further research is needed to better clarify these relationships and to incorporate multiple items within each latent construct in order to more accurately represent mental health problems.

Second, our structural equation modelling imposes a temporal ordering of variables with ACEs preceding family resilience and mental health outcomes. However, this approach does not confirm causality due to the cross-sectional design of the survey data. Particularly, causal mediation claims typically require longitudinal or experimental designs (Maxwell & Cole, 2007). Given our cross-sectional design, the indirect effects identified in this study should be carefully interpreted as associational, not causal. Future research is encouraged to employ a longitudinal design to more firmly establish causal relationships.

Third, the operationalization of externalizing and internalizing problems may not precisely capture the complexity of each mental health condition. Certain conditions, particularly ADHD, are more complex and heterogeneous than can be fully explained by this dichotomous classification (i.e., internalizing vs. externalizing problems). For example, other subtypes of ADHD (i.e., predominantly inattentive and combined with hyperactive-impulsive types) might not be explained by externalizing problem category. Although prior research reported externalizing trajectories between ADHD and conduct problems (Atherton et al., 2020), ADHD can also be categorized as one of neurodevelopmental disorders (Thapar et al., 2017). Future research can benefit from more delicate operationalization and classification of mental health constructs by employing multi-indicator or pattern-based approaches.

Fourth, although a high-order CFA is conceptually demonstrated a good model fit and is supported by prior research on hierarchical structures of youth mental health (Cosgrove et al., 2011), additional theoretical testing is warranted to compare and contrast alternative measurement models of mental health items, such as a single-factor model and a bifactor model, to more rigorously evaluate the dimensional structure of youth mental health outcomes. Further research should test higher-order CFA models to confirm the validity of this mental health model (Brown, 2015; Dunn & McCray, 2020).

Fifth, while threshold-based approach adopted in this study was well-suited to identifying youth with clinically significant mental health problems, this method necessarily collapses variability in symptom severity both within and across diagnostic thresholds. As a result, more subtle gradations in risk, particularly among youth with subthreshold symptoms, may not have been fully captured. Future research could complement threshold-based classifications with continuous or dimensional measures of psychopathology to examine whether associations with ACEs vary across the full severity spectrum, rather than only the point of clinical threshold.

Sixth, although the ACEs items reflect core aspects of the construct, they still lack sufficient representation of certain types of adversity, particularly those related to identity-based discrimination (e.g., disability, sexual discrimination), sexual/physical/emotional abuse, and neglect experiences. The roles and impacts of these types of ACEs should be further investigated within this model in future research.

Finally, while our study focuses on family-level factors, such as challenges and resilience, incorporating a broader context that includes individual and environmental factors (e.g., coping mechanisms, SES, peer and social support) would improve the findings. This could involve considering intersecting identities, community environments, and cultural considerations to better explain this study model. Future research is recommended to further explore how the combined effects of ACEs and various levels of individual and environmental factors shape and potentially buffer the development of mental health concerns in youth.

## Conclusion

Using nationally representative data and sample of youth aged 6–17 years, our findings suggest that family resilience accounts for a significant indirect effect in the relationship between ACEs and mental health outcomes. This finding indicates that ACEs are associated with lower family resilience, potentially leading to adverse mental health outcomes, including ADHD, behavioral problems, anxiety, and depression. Focusing on a family’s strengths and building upon them in intervention efforts can help mitigate some of the challenges linked to ACEs. Tailoring resilience-focused interventions to the family’s specific context can provide critical support systems and reduce the risk of mental health concerns among youth.

## Supplementary Information

Below is the link to the electronic supplementary material.

Supplementary Material 1

## Data Availability

The data that support the findings of this study are openly available in United States Census Bureau - NSCH Datasets at https://www.census.gov/programs-surveys/nsch/data/datasets.2022.html#list-tab-491554181.

## Declarations

### AI Statement

An AI-assisted tool was used solely for language editing and grammar checking.
