# Supplementary material for: The Impact of Adverse Childhood Experiences on Youth’s Mental Health: What is the Role of Family Resilience?
Source: J Child Adolesc Trauma. 2026 Aug 27;19(4):2587–99. doi: 10.1007/s40653-026-00966-4 (PMC13737346; doi:10.1007/s40653-026-00966-4)
Supplement: Supplementary file 1 [file 40653_2026_966_MOESM1_ESM.docx]

| **Supplement 1**  **Table S1.** *Item Descriptions and Factor Loadings for Study Variables* | | | | | | |
| --- | --- | --- | --- | --- | --- | --- |
| Variables | Description | | | Unstandardized  Factor Loadings (B) | Standardized  Factor Loadings  (β) | |
| ACE1  (Economic hardship) | Child experienced hard to cover basics like food or housing since this child were born on this child’s family’s income | | | 1.000 | 0.520 | |
| ACE3  (Divorce) | Child’s experience for parent or guardian divorced or separated | | | 1.286 | 0.668 | |
| ACE4  (Death) | Child’s experience for parent or guardian died | | | 0.723 | 0.376 | |
| ACE5  (Jail) | Child’s experience for parent or guardian served time in jail or prison | | | 1.519 | 0.790 | |
| ACE6  (Domestic violence) | Child saw or heard parents or adults slap, hit, kick, and punch one another in the home | | | 1.682 | 0.874 | |
| ACE7  (Neighborhood violence) | Child was a victim of violence or witnessed violence in this child’s neighborhood | | | 1.510 | 0.785 | |
| ACE8  (Mental health) | Child lived with anyone who was mentally ill, suicidal, or severely depressed | | | 1.463 | 0.761 | |
| ACE9  (Drug use) | Child lived with anyone who had a problem with alcohol or drugs | | | 1.607 | 0.836 | |
| ACE10 (Discrimination) | Child was treated or judged unfairly because of this child’s race or ethnic group | | | 0.707 | 0.368 | |
| FAM1 | Facing Problems - How Often Talk Together | | | 1 | 0.829 | |
| FAM2 | Facing Problems - How Often Draw on Strengths | | | 1.015 | 0.873 | |
| FAM3 | Facing Problems - How Often Stay Hopeful | | | 0.810 | 0.747 | |
| FAM4 | Facing Problems - How Often Work Together | | | 1.054 | 0.891 | |
| Category | Subcategory | B | β | Observed | B | β |
| Mental health outcomes | Internalizing Problems | 1 | 0.863 | Depression | 1 | 0.961 |
|  |  |  |  | Anxiety | 0.916 | 0.880 |
|  | Externalizing Problems | 0.754 | 0.744 | ADHD | 1 | 0.837 |
|  |  |  |  | Behavioral | 1.097 | 0.919 |
